# CCCNet: Criss-cross attention enhanced cross layer refinement network for lane detection in complex scenarios

**DOI:** 10.1371/journal.pone.0321966

**Published:** 2025-05-13

**Authors:** Bo Liu, Haoran Sun, Zijie Chen

**Affiliations:** 1 School of Computer Science and Artificial Intelligence, Chaohu University, Chaohu, China; 2 School of Computer Science and Engineering, Macau University of Science and Technology, Macau, Macau; Van Lang University: Truong Dai hoc Van Lang, VIET NAM

## Abstract

Lane detection plays a crucial role in autonomous driving systems by enabling vehicles to comprehend road structure and ensure safe navigation. However, the current performance of lane line detection models, such as CCNet, exhibits limitations in handling difficult driving conditions like shadows, nighttime, no lines,and dazzle, which significantly impact the safety of autonomous driving. In addition, due to the lack of attention to both the global and local aspects of road images, this issue becomes even more pronounced. To address these challenges, we propose a novel network architecture named Criss-Cross Attention Enhanced Cross-Layer Refinement Network (CCCNet). By integrating the strengths of criss-cross attention and cross-layer refinement mechanisms, CCCNet effectively captures long-range dependencies and global context information from the input images, leading to more reliable lane detection in complex environments. Extensive evaluations on standard datasets, including CULane and TuSimple, demonstrate that CCCNet outperforms CLRNet and other leading models by achieving higher accuracy and robustness, especially in challenging scenarios. In addition, we publicly release our code and models to encourage further research advancements in lane detection technologies at https://github.com/grass2440/CCCNet.

## Introduction

**Fig 1:**
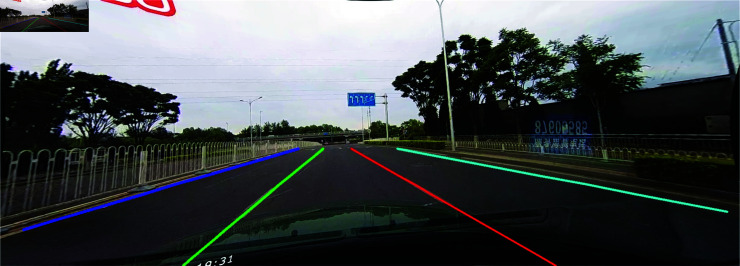
An example of lane detection.

**Fig 2:**
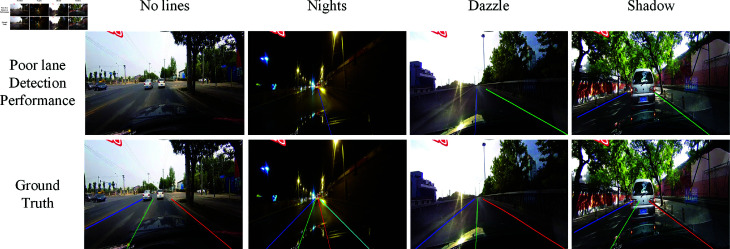
Challenging scenarios with poor lane line detection performance.

Lane detection is a computer vision technique that identifies and tracks lane markings on roads. The process involves analyzing images captured by cameras mounted on vehicles to recognize lane boundaries, which may be solid, dashed or partially obscured. An example of lane detection is shown in Fig 1, where four different colors represent the four lane lines on the road. Despite significant advancements in lane detection technology, challenges continue to persist in specific scenarios, such as conditions influenced by shadows, absence of visible lines, nighttime driving, and situations with glaring lights. As illustrated in Fig 2, poor lane detection performance is evident in various challenging scenarios, including instances where lane markings are not detected, and in some cases, no lanes are detected at all. This figure serves as a useful reference, as the second row shows the ground truth, providing a benchmark for evaluating the performance of lane detection systems in these difficult contexts. To address these challenges, it is imperative to enhance the performance of lane detection systems in these difficult contexts. The present research seeks to build upon existing high-performance models, exploring innovative methods and techniques to improve the accuracy and reliability of lane detection. By concentrating on these critical scenarios, the aim is to facilitate safer driving experiences across all conditions.

Current high-precision lane detection models are predominantly dependent on artificial neural networks. Among these, CLRNet [1] stands out as a prominent model, achieving excellent lane detection performance, particularly on datasets such as CULane where it has exhibited cutting-edge performance. However, we contend that there remains potential for enhancement in CLRNet’s performance in certain challenging scenarios within the CULane dataset, including shadows, absence of lines, nighttime conditions, and dazzling effects. We posit that augmenting the global attention mechanism can bolster lane detection capabilities in intricate scenarios. This study introduces a streamlined and proficient global attention module, namely the Recurrent Criss-Cross Attention (CCA) module [2], into the CLRNet framework to amplify its lane detection accuracy.

The primary contributions of this study are three-fold:

(1) Review and Proposal of CCCNet: Conducted a comprehensive review of current advancements in lane detection technology, identifying areas for further optimization, particularly in challenging scenarios such as darkness, glare, and shadows. Introduced CCCNet, an efficient method combining a cross-attention mechanism with the CLRNet architecture to improve lane detection accuracy across diverse scenarios, marking the first application of such an attention mechanism in lane detection models.
(2) Exploration and Application of Global Attention Mechanism: We identified a suitable global attention mechanism (Recurrent Criss-Cross Attention) for current SOTA lane detection models and integrated it into the SOTA model. This led to the development of the Criss-Cross CLRNet (CCCNet), marking the first application of this attention mechanism in lane detection.
(3) Experiments and Open-Source Contribution: Trained and evaluated CCCNet on the CULane and TuSimple datasets, achieving superior performance compared to state-of-the-art methods. Additionally, we have made our code publicly accessible at the following https://github.com/grass2440/CCCNet.git.

## Related work

Lane detection has undergone significant advancements in recent years, primarily driven by deep learning and artificial intelligence techniques. Early lane detection methods predominantly relied on classical image processing techniques such as Hough transforms, color segmentation, and edge detection. In recent years, the rapid advancement of artificial intelligence has substantially revolutionized the approach to lane detection.

Segmentation-based approaches formulate lane detection as a semantic segmentation problem, classifying each pixel in an image as belonging to a lane or not. Pan *et al*. (2018) introduced SCNN(Spatial Convolutional Neural Network) [3], which extends traditional convolutional neural networks (CNNs) by adding a spatial convolution operation. This operation enables message passing between pixels across rows and columns, capturing long-range dependencies along the lane lines. Zheng *et al*. (2021) proposed RESA (Recurrent Feature-Shift Aggregator) [4], which enhances global context aggregation by recurrently shifting feature maps in four directions (left, right, up, down) and aggregating them. This mechanism allows the network to efficiently capture global contextual information without the heavy computational costs associated with traditional recurrent neural networks. Yoo *et al*. (2020) simplified lane detection by performing row-wise classification. The proposed network E2E (End-to-End Lane Marker Detection via Row-wise Classification) [5] predicts lane positions at each row of the image, reducing complexity and enabling real-time performance while maintaining competitive accuracy.

Proposal-based methods detect lanes by generating proposals or using predefined anchors, keypoints, or polynomial fitting. Philion (2019) introduced FastDraw [6], which models lanes as sequences of points and employs an LSTM-based decoder for sequential prediction. This method effectively captures global lane structures and addresses the variability in lane appearances, including rare and complex lane patterns, FastDraw also employs attention mechanisms within a sequential prediction framework to address rare and complex lane patterns, effectively capturing long-range dependencies. Ko *et al*. (2021) formulated lane detection as a keypoint estimation and clustering task. The proposed network PINet (Point Instance Network) [7] predicts keypoints along lane lines and uses embedding vectors to cluster points belonging to the same lane. Qu *et al*. (2021) proposed FOLOLane (Focus on Local) [8], a bottom-up approach that detects lane markers via keypoint estimation. By focusing on local regions and detecting keypoints that are then connected to form lane lines, FOLOLane effectively handles occlusions and varying lighting conditions. Tabelini *et al*. (2020) proposed PolyLaneNet [9], which models lanes as parametric curves and predicts polynomial coefficients. This allows efficient handling of lanes with various shapes and curvatures, enhancing the model’s capability to generalize across different road geometries.

Attention and context-aware methods incorporate attention mechanisms and leverage global contextual information to enhance lane detection, particularly in complex environments. Tabelini *et al*. (2021) introduced an anchor-based approach combined with attention mechanisms. LaneATT [10] applies self-attention over predefined anchors to enhance feature representation and improve detection accuracy, achieving state-of-the-art results on several benchmarks. Liu *et al*. (2023) proposed NLNet (Non-Local Neural Network for Lane Detection) [11] which integrates non-local neural networks to enhance long-range dependencies in lane detection. By incorporating non-local operations, NLNet captures global contextual information and models the relationships between distant pixels, improving the network’s ability to detect lanes in challenging scenarios with occlusions and complex road geometries. Abualsaud *et al*. (2021) presented LaneAF (Lane Association Fields) [12], which predicts lane association fields to capture the relationships between pixels and lane instances. Su *et al*. (2021) proposed SGNet (Structure Guided Network) [13], which incorporates structural guidance into the detection process. The model predicts vanishing points and utilizes structural priors to inform lane detection, enhancing performance in challenging conditions such as curves and complex road geometries. Hou *et al*. (2019) proposed ENet-SAD (Self-Attention Distillation for Lane Detection) [14], which integrates self-attention distillation into a lightweight network. By distilling attention maps from a teacher network, ENet-SAD improves the feature representation capacity of a smaller student network, achieving high accuracy with reduced computational cost. Liu *et al*. (2020) introduced LSTR (Lane Shape Prediction with Transformers) [15], which applies transformer architectures to lane detection. By treating lane detection as a shape prediction problem and using self-attention mechanisms, LSTR (Lane Shape Prediction with Transformers) effectively models long-range dependencies and global context.

Recent advancements in lane detection have focused on improving accuracy and robustness in complex scenarios, such as night, dazzle, and shadow. Techniques like multi-scale feature fusion, as demonstrated in CFRNet: Road Extraction in Remote Sensing Images Based on Cascade Fusion Network [16], have shown significant promise in capturing both local and global context for linear structure detection. CFRNet’s cascade fusion mechanism effectively integrates features from different network levels, making it particularly suitable for handling challenges like varying road widths and complex backgrounds. Similarly, in lane detection, multi-scale feature fusion can enhance the model’s ability to detect lanes in diverse and challenging conditions. Another critical area of research is efficient data utilization, particularly in reducing the dependency on large annotated datasets. Active Learning for Deep Visual Tracking [17] addresses this challenge by prioritizing the most informative samples for labeling, thereby reducing annotation costs while maintaining model performance. This approach is highly relevant for lane detection, where obtaining large-scale annotated datasets is often costly and time-consuming. By incorporating active learning techniques, future lane detection models could achieve comparable accuracy with significantly less labeled data. Finally, temporal modeling has emerged as a powerful tool for handling dynamic environments, as demonstrated in Aligned Spatial-Temporal Memory Network for Thermal Infrared Target Tracking [18]. This work leverages spatial-temporal memory networks to store and retrieve relevant features over time, improving tracking accuracy in challenging conditions like low visibility and dynamic backgrounds. Such techniques could be adapted for lane detection to handle temporal variations, such as changes in lane markings due to weather or lighting, further enhancing model robustness. In 2019, Neven *et al*. [19]proposed an instance segmentation-based approach for end-to-end lane detection, providing a new perspective on the task (Neven *et al*., 2019). Building on this, Qin *et al*. (2020) [20]introduced an ultra-fast structure-aware deep lane detection method, significantly improving detection speed through an efficient design. In 2022, Liu *et al*. [21]proposed CondLaneNet, a top-down lane detection framework based on conditional convolution, which improved accuracy through a hierarchical design.Then, In 2024, CLRerNet [22] (Hiroto Honda *et al*.) introduces LaneIoU, aligning confidence scores with IoU using local lane angles, achieving state-of-the-art F1 scores. Also in 2024, GSENet [23] (Junhao Su *et al*.) tackles complex scenarios with a Global feature Extraction Module (GEM) and novel loss functions like Angle Loss, enhancing global context understanding. These works collectively highlight the evolution of lane detection techniques, from instance segmentation and attention mechanisms to Transformers and dynamic feature fusion, providing a strong foundation for the development of our CCCNet model.

## Motivation

In the context of lane detection, particularly in complex scenarios such as shadows, nighttime, no lanes, and dazzle, models encounter numerous challenges. In environments obscured by shadows, intense light and shadow patterns can obscure lane markings, potentially leading to model misjudgments or omissions. During nighttime conditions, lane markings might be invisible, requiring the model to rely on its inferential capabilities. In scenarios where lane markings are absent, the model must infer potential lane locations based on surrounding environmental cues. Dazzle scenarios, characterized by intense light sources, may blur or entirely obscure lane markings. Hence, a variety of scenarios present significant challenges to road detection methods. The critical issue at hand is how to mitigate and address the adverse impact of interference information on road identification across different scenes.

Attention mechanisms, which mimic the human visual system’s ability to focus on salient regions, have been increasingly integrated into various domains of object detection. This mechanism enables models to concentrate on areas rich in information during image processing, thereby enhancing the accuracy and efficiency of detection. Within the context of object detection tasks, attention mechanisms facilitate improved recognition and localization of target objects, particularly in scenarios with complex backgrounds and multiple targets, where they significantly boost model performance. Through the application of attention mechanisms, models can autonomously learn the critical features of targets while disregarding irrelevant background noise, leading to superior performance in a range of application scenarios including pedestrian detection, vehicle detection, and facial recognition. The self-attention mechanism involves the model initially calculating dot product similarities between features to determine dependencies, with features exhibiting higher similarity exerting a greater impact on output generation. This process assists the model in identifying crucial features within complex scenarios. The model performs a weighted summation of input features through the generated attention weights, ensuring that the final feature representation is not solely dependent on a single feature but integrates influences from multiple features, thereby achieving a global contextual information synthesis.

The incorporation of attention mechanisms in road detection tasks represents a significant breakthrough, markedly enhancing the model’s capability to detect road features. In environments where the visibility of lane lines is compromised, for example in conditions of shadow, the attention mechanism enables the model to identify and prioritize features most correlated with lane lines, thereby enhancing their weightage. For instance, when the model detects shadows near lane lines, the attention mechanism guides the model to concentrate on the inherent characteristics of the lane lines, rather than the shadows. In conditions where lane lines are not visible, such as at night, the model relies on environmental features, such as streetlights and vehicle lights, for inference. The attention mechanism integrates global contextual information, enabling the model to deduce probable lane positions based on surrounding environmental cues in the absence of direct lane line data. In scenarios without lane markings, the model depends on environmental features for judgment. The attention mechanism weights these surrounding features, assisting the model in determining potential lane line locations. By focusing on surrounding features, the model can infer possible navigational paths even in the absence of lane lines. In dazzle scenarios, strong light sources might cause lane lines to appear blurred or completely hidden. In such cases, the attention mechanism dynamically adjusts focus, augmenting features unaffected by glare. Through feature weighting, the model can disregard strong light interferences, concentrating instead on other vital information. However, traditional attention computations are resource-intensive. Consequently, in this study, we adopt a computationally efficient attention module and seamlessly integrate it into the currently commonly used road detection model, which can improve the detection accuracy with only minor modifications.

## The proposed model: CCCNet

The complexity and variability of driving scenes exacerbate the challenges in lane detection. As mentioned above, In intricate scenarios such as shadows, nighttime, absence of lines, and highlights, lane detection models devoid of global information exhibit subpar performance. Selection of an effective inspection network skeleton is a critical operation for road inspection tasks. Distant pixels often exhibit certain correlations, by integrating this contextual information, more effective feature maps can be generated, which in turn enhances the model’s ability to understand semantics. Traditional CNN models are constrained by the kernel window size and cannot estimate global information. ASPP (Atrous Spatial Pyramid Pooling) employs parallel convolution layers with different dilation rates to extract features at various scales, thereby capturing multi-scale contextual information. PPM (Pyramid Pooling Module) utilizes pooling layers of different scales (such as 1×1,2×2, 3×3, 6×6) to capture multi-scale information. These pooling layers extract contextual features of varying sizes, and PPM upsamples and fuses feature maps from different scale pooling layers, generating a rich contextual information feature map. However, dilated convolutions fail to generate dense contextual information [2]. Moreover, pooling-based methods aggregate contextual information in a non-adaptive manner and assume all image pixels are equally important, failing to meet the varying context dependencies required by different pixels. Although the Non-local attention mechanism effectively addresses these issues and has been integrated into lane detection method to improve performance, the Non-local module requires vast attention feature maps to represent pairwise relationships between pixels, significantly increasing computational demands.

To develop a more effective road detection approach, we have selected CLRNet as our backbone network. Utilizing the Feature Pyramid Network (FPN) structure, CLRNet optimally integrates high-level semantic features with low-level spatial details, enabling robust detection of objects across different scales. Specifically, the construction of the feature pyramid can be mathematically expressed as:

$$P_{l}=Top\text{-}Down(C_{l})+Up\text{-}Sample(P_{l+1})$$ (1)

where *P*_*l*_ represents the feature map at scale *l*, and *C*_*l*_ denotes the convolutional features at scale *l*. The Top-Down operation fuses features from higher levels while the Up-Sample operation enlarges the lower-level feature maps to match the dimensions of higher-level ones. The pyramid structure allows the network to capture features at multiple scales, providing a comprehensive understanding of contextual information. However, this approach still lacks an effective global attention mechanism.

Building upon the previous analysis, we identified two key directions for improvement: First, our comprehensive investigation of state-of-the-art lane detection models from recent years revealed that strengthening the global attention mechanism significantly enhances detection performance; second, we explored the integration of the Criss-Cross attention module with the advanced CLRNet framework. Based on these findings, we propose CCCNet (Criss-Cross Attention Enhanced Cross-Layer Refinement Network) as an innovative solution.

### The highly computationally efficient global attention mechanism

This section elucidates the manner in which Criss-Cross Attention effectively captures global contextual information by comparing and analysing it with Nonlocal. Despite both utilizing global attention mechanisms, the Criss-Cross Attention exhibits superior performance compared to the non-local approach [11, 24]. The nonlocal module gathers global features by calculating relationships among all pixels in the input feature map. Specifically, it calculates the similarity between each pixel and all others via an attention mechanism and then uses these similarities to create a new feature representation, incorporating contextual information from all pixels into each pixel’s features. This allows the nonlocal module to effectively capture relationships between any two pixels, improving the model’s understanding of global information. The nonlocal attention mechanism is mathematically expressed as:

$$Non\text{-}local(X)=\frac{1}{C(X)}\sum_{i,j}f(x_{i},x_{j})g(x_{j})$$ (2)

In this study, *X* denotes the input feature map, while $f(x_{i},x_{j})$ represents the function employed to calculate the similarity between pixels *x*_*i*_ and *x*_*j*_. This is typically accomplished through the use of a dot-product or an alternative similarity measure. The term *g*(*x*_*j*_) signifies the feature at pixel *x*_*j*_. Finally, *C*(*X*) is a normalization factor that ensures the sum of attention weights is equivalent to one.

**Fig 3:**
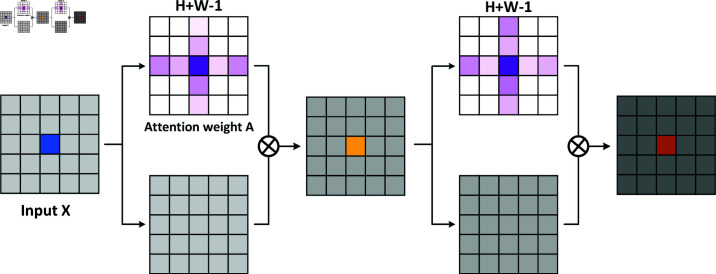
The overall structural of the criss-cross attention mechanism.

In contrast, the Criss-Cross Attention mechanism calculates attention separately along the row and column dimensions of the image, thereby enabling the capture of global context through two consecutive attention operations. The overall structural of the Criss-Cross Attention mechanism is shown in Fig 3. This row and column-wise attention mechanism simplifies computational complexity while effectively maintaining the ability to capture global dependencies. The Criss-Cross Attention mechanism operates as follows:

$$Criss\text{-}Cross\;Attention(X)=\sum_{i,j}A_{i,j}X_{j}$$ (3)

In this formula, *X* denotes the input feature map, and *A*_*i*,*j*_ is the attention weight between pixel *i* and pixel *j* in the row or column direction. The feature at position *j* is represented by *X*_*j*_. By applying these row and column attention weights to the input feature map, Criss-Cross Attention generates a new feature map that captures contextual information from both the horizontal and vertical directions. This dual-row-column approach has been shown to significantly reduce computational complexity from *O*(*N*^2^)(in the non-local approach) to *O*(*N*), thus rendering it more computationally efficient. To illustrate this, consider a 1024 x 1024 image; each pixel now only needs to compute 1024 relationships for its row and 1024 relationships for its column, summing to a total of 2048 computations per pixel. This reduction in complexity has been shown to enhance efficiency, particularly in the context of high-resolution images.

Furthermore, Criss-Cross Attention has been demonstrated to outperform non-local attention with respect to the capture of pertinent contextual information, particularly in tasks such as lane detection, where spatial relationships along rows and columns are of critical importance. In urban scenes, where pedestrians and vehicles are frequently aligned vertically or horizontally, Criss-Cross Attention adeptly integrates this information by focusing on relevant features within the same row or column while ignoring irrelevant ones. Conversely, the non-local mechanism’s indiscriminate aggregation of global context can result in the inclusion of irrelevant objects, such as buildings, in the feature representations of nearby objects, such as pedestrians, potentially leading to a degradation in segmentation accuracy. Thus, when considering both computational efficiency and task-specific requirements, Criss-Cross Attention offers distinct advantages for lane detection tasks, providing a more accurate and resource-efficient solution.

### CCCNet

**Fig 4:**
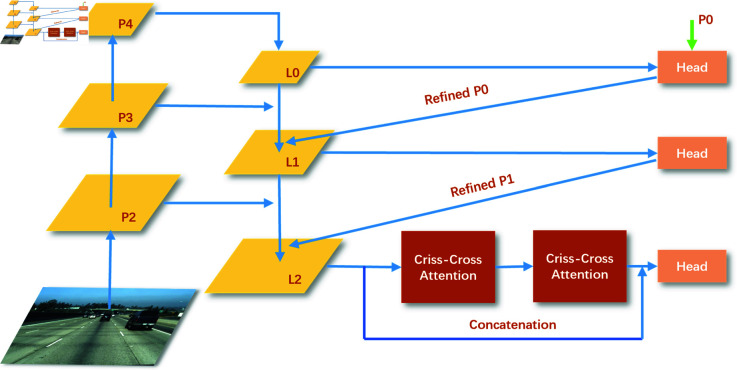
The overall structure of the CCCNet.

As illustrated in Fig 4, Criss-Cross Attention has been integrated into CLRNet, particularly within the Feature Pyramid Network of CLRNet. It should be noted that a single Criss-Cross Attention operation is capable of capturing information from only the same row and column for each pixel. CCCNet employs two consecutive Criss-Cross Attention operations to capture information from all pixels, thereby achieving global context [2]. The CLRNet model utilizes multi-scale feature fusion, progressively refining features through a top-down pathway, with the capture of global information primarily conducted via the Head. The argument is made that the current capture of global contextual information is inadequate; therefore, the primary purpose of introducing the Criss-Cross Attention module is to enhance the model’s ability to capture global contextual information. The integration of this module is expected to result in the establishment of a more comprehensive global receptive field, thereby facilitating the capture of long-range dependencies between features.

In this study, we implement the Criss-Cross Attention module following the L2 layer. While this attention mechanism could alternatively be applied after the L0 or L1 layers, such multi-layer deployment would significantly increase model complexity, demanding greater computational resources during training and potentially compromising inference efficiency. Since the L2 features already integrate high-level semantic information with low-level detail, they are more effective in supporting the modeling of global context compared to L0 and L1. After a comprehensive analysis, we decided to place the Criss-Cross Attention module after L2, allowing us to effectively capture global contextual information while maintaining computational efficiency, thereby improving the accuracy of lane detection.

In CCCNet, the input road surface image undergoes multi-scale feature extraction through the Backbone network (such as ResNet [25] and DLA34 [26]), resulting in the feature pyramid P2, P3, P4. Subsequent feature fusion and refinement are performed: high-level feature P4 is upsampled and fused with P3 to obtain L1. L1 is then upsampled and fused with P2 to obtain L2. L0, L1, L2 constitute the new feature pyramid, integrating information from different scales. The L2 feature undergoes two consecutive Criss-Cross Attention modules to capture global contextual information and enhance feature expressiveness. The outputs of the three Heads correspond to different feature levels: Head 1 (connected to L0) produces an initial prediction using the highest-level feature (L0), containing global semantic information but possibly lacking precise localization. Head 2 (connected to Refined P0) takes the refined feature from P0 combined with L1 as input, producing P1, which optimizes upon P0 and improves localization precision. Head 3 (connected to Refined P1) integrates the L2 feature, the refined feature from P1, and the effectively captured global features from Criss-Cross Attention, producing the final detection result P2.

## Training and testing

### Dataset

To maintain consistency with the dataset used in Literature [1] and to facilitate comparison, this study was conducted using two datasets: CULane and Tusimple.

The CULane dataset, tailored for lane detection tasks, is comprehensive and large-scale. It contains over 133,000 images depicting diverse road conditions in both urban and rural settings. The dataset stands out for its inclusion of complex scenarios such as night driving, shadows, absent lane markings, arrows, and glare. Each image in this dataset is of high resolution (1640×590), enabling detailed feature extraction essential for accurate lane detection. The dataset provides pixel-level annotations for lane markings, essential for training deep learning models to precisely identify and track lanes. These annotations support the development and evaluation of algorithms designed to perform reliably in real-world scenarios.

Scenario Types in the CULane Dataset:

1. Normal: This is a typical lane detection scene with clear lane markings and a clean background, suitable for assessing basic model performance.
2. Crowded: Characterized by dense vehicle traffic that may obscure lane markings, this scene increases detection complexity. Models need to handle interference from multiple vehicles to accurately identify lane lines.
3. Dazzle: Intense light reflections or direct sunlight cause glare in this scene, potentially obscuring lane markings. This scenario tests model performance under high contrast and changing light conditions.
4. Shadow: This scene includes pronounced shadows, possibly from buildings, trees, or other objects, which can interfere with the visibility of lane markings and increase detection difficulty.
5. No Line: Lacking clear lane markings, models must rely on other contextual information like road shape or environment to infer lane positions.
6. Arrow: This scene contains directional arrows indicating the driver’s direction. Models need to account for the relationship between lane markings and arrows to accurately identify lanes.
7. Curve: Containing road curves where the shape of lane lines may vary, models must adapt to curve shapes for correct detection.
8. Cross: Involving intersections where lane markings may become complicated, models need to recognize lane lines in multiple directions.
9. Night: Images taken under night conditions have reduced visibility and less distinct lane markings. Models must effectively detect lane lines in low-light environments.

We present the performance of the models in each scenario within the experimental results.

The Tusimple dataset is another widely utilized resource for lane detection, specifically designed to focus on highway driving scenarios. It includes a total of 6,408 training images and 2,782 test images. Each image is typically of resolution 1280×720, offering a balance between detail and computational efficiency. This resolution allows for effective feature extraction while maintaining manageable processing requirements. Annotations are provided in the form of polylines that represent lane centerlines. These annotations are crucial for training models to understand lane shapes and continuity, enabling accurate lane detection and tracking. The dataset’s focus on highway scenarios makes it ideal for developing systems aimed at autonomous driving and advanced driver assistance systems. To further validate the superior performance of the CCCNet model, we conducted tests on the Tusimple dataset and compared the results with those obtained from the CLRNet model.

### Hyperparameter setting and training

The training and testing environment consists of the following specifications: Ubuntu version 18.04.6 LTS, CUDA version 11.3, cuDNN version 8.2.0.5, Python version 3.8.18, and PyTorch version 1.8.0.

When training the CCCNet model on the CULane dataset, the batch size was set to 24. The learning rate (lr) was set to 1.0e^−3^ for training from epoch 0 to epoch 35, and then it continued with 1r=0.6e^−3^ for an additional 4 epochs starting from epoch 36. For the Tusimple dataset, when using resnet18 as the backbone, the batch size was set to 40 and the learning rate (lr) was 1.0e^−3^, with training conducted over 71 epochs. With resnet34 as the backbone, the batch size was adjusted to 32 and the learning rate (lr) was set to 0.8e^−3^, resulting in 72 epochs of training. When employing resnet101 as the backbone, the batch size was reduced to 10 and the learning rate (lr) was lowered to 0.3e^−3^,leading to a total of 82 epochs of training.

## Experiment

### Experiment metrics

In this study, we adhere to industry standards by using the F1 score as the primary evaluation metric. The formula for calculating the F1 score is as follows:

$$F1=\frac{2\times (precision\times recall)}{\left(precision+recall\right)}$$ (4)

Precision measures how many of the positive predictions made by a model are correct, while recall measures how many of the positive examples in the data are correctly identified by the model. The formulas for calculating Precision and Recall are provided below.

$$Precision=\frac{TP}{\left(TP+FP\right)}$$ (5)

$$Recall=\frac{TP}{\left(TP+FN\right)}$$ (6)

In various contexts, Positive Predictions denote the outcomes from a model that are identified as belonging to the positive category, such as identifying a specific object. These are the outputs of the model suggesting that it identifies the presence of a target within a certain location or region. For example, in lane detection, when the model identifies and marks a lane line, this is deemed a positive prediction. Positive Examples, on the other hand, are the authentic instances of the positive category within the dataset. In essence, positive examples are the actual objects that the model is intended to recognize. For instance, in a lane detection dataset, all the actual lane lines are categorized as positive examples. A True Positive (TP) is when the model accurately predicts the positive category. This means that the model not only forecasts the presence of an object (a positive prediction), but this forecast aligns with the reality. For example, if the model successfully identifies a real lane line, that identification is a True Positive. Conversely, if the model inaccurately marks a non-existent lane line, this would not qualify as a True Positive but could be classified as a False Positive (FP). A False Negative (FN) occurs when the model mistakenly predicts the negative category. This implies that the model perceives an object as absent (thereby making a negative prediction), when in fact, the object is present. For lane detection, if the model fails to identify a lane line that is actually there and does not mark it, this is considered a False Negative. This typically suggests that the model’s predictions are too cautious, missing targets that should have been identified.

In addition to F1, this study employs mF1, F1@50, and F1@75 as evaluation metrics. F1@50 represents the value of F1 when the IoU threshold is 50%, while F1@75 represents the value of F1 when the IoU threshold is 75%. IoU (Intersection over Union) is a common metric used to assess the degree of overlap between two regions (typically the predicted bounding box and the ground truth bounding box). The formula for calculating IoU is given below,

$$\text{IoU}=\frac{\text{(Area of Overlap)}}{\text{(Area of Union)}}$$ (7)

where Area of Overlap represents the area where the predicted bounding box and the ground truth bounding box overlap, and Area of Union represents the total area covered by both the predicted and ground truth bounding boxes (i.e., the area of their union). Referencing the CLRNet detection metric, mF1 is defined as:

$$mF1=\frac{\left(F1@50+F1@55+...+F1@95\right)}{10}$$ (8)

where *F*1@50,*F*1@50,...,*F*1@95 are F1 metrics when IoU thresholds are 50%, 55%, ..., 95% respectively.

### Experiment result

The original CLRNet model, as presented in Reference [1], was evaluated with four distinct backbone architectures: ResNet18, ResNet34, ResNet101, and DLA34. Experimental results demonstrated that DLA34 yielded optimal detection performance on the CULane dataset. To ensure fair comparison on the CULane benchmark, our CCCNet model adopts the same DLA34 backbone. This consistency extends to the Tusimple dataset, where CCCNet maintains architectural parity with CLRNet by utilizing identical backbone networks for comparative analysis.

Table 1 presents the test results of our proposed CCCNet compared to other mainstream lane detection models, including CLRNet, on the CULane dataset. Bolded values indicate optimal performance. The evaluation metrics include mF1, F1@50 for the entire test set, F1@75 for the entire test set, and F1@50 for each scenario in CULane, with only false positives (FP) shown for the “Cross” scenario. The data in the table clearly demonstrates that prior to the introduction of CCCNet, CLRNet exhibited the best performance. Additionally, after the introduction of CCCNet, it achieved state-of-the-art results, showing not only a slight advantage in mF1 but also significant improvements in challenging scenarios. The specific experimental results for different scenarios under the CULane dataset are shown in Table 2 In the Dazzle scenario, lane detection performance improved from 75.30 to 77.06, while in the Night scenario, it increased from 75.37 to 76.01. The GFlops of our proposed CCCNet is 18.5, and when measured in GFlops and rounded to one decimal place, it is the same as the GFlops of the original SOTA model. This indicates that the increase in computational load is minimal, at least when rounded to one decimal place, they are identical.

**Table 1:** Comparison of experimental results on CULane dataset.

Method	Backbone	GFlops	mF1	F1@50	F1@75
SCNN	VGG16	328.4	38.84	71.60	39.84
RESA	ResNet34	41.0	-	74.50	-
RESA	ResNet50	43.0	47.86	75.30	53.39
FastDraw	ResNet50	-	-	-	-
E2E	ERFNet	-	-	74.00	
UFLD	ResNet18	8.4	38.94	68.40	40.01
UFLD	ResNet34	16.9	-	72.30	-
PINet	Hourglass	-	46.81	74.40	51.33
LaneATT	ResNet18	9.3	47.35	75.13	51.29
LaneATT	ResNet34	18.0	49.57	76.68	54.34
LaneATT	ResNet122	70.5	51.48	77.02	57.50
LaneAF	ERFNet	22.2	48.60	75.63	54.53
LaneAF	DLA34	23.6	50.42	77.41	56.79
SGNet	ResNet18	-	-	76.12	-
SGNet	ResNet34	-	-	77.27	-
FOLOLane	ERFNet	-	-	78.80	-
CondLane	ResNet18	10.2	51.84	78.14	57.42
CondLane	ResNet34	19.6	53.11	78.74	59.39
CondLane	ResNet101	44.8	54.83	79.48	61.23
CLRNet	DLA34	18.5	55.64	80.47	62.78
CCCNet(ours)	DLA34	18.5	55.67	80.31	62.86

**Table 2:** Comparison of experimental results on CULane dataset.

Method	Backbone	Normal	Crowded	Dazzle	Shadow	No line	Arrow	Curve	Cross	Night
SCNN	VGG16	90.60	69.70	58.50	66.90	43.40	84.10	64.40	1990	66.10
RESA	ResNet34	91.90	72.40	66.50	72.00	46.30	88.10	68.60	1896	69.80
	ResNet50	92.10	73.10	69.20	72.80	47.70	88.30	70.30	1503	69.90
E2E	ERFNet	91.00	73.10	64.50	74.10	46.60	85.80	71.90	2022	67.90
UFLD	ResNet18	87.70	66.00	58.40	62.80	40.20	81.00	57.90	1743	62.10
	ResNet34	90.70	70.20	59.50	69.30	44.40	85.70	69.50	2037	66.70
PINet	Hourglass	90.30	72.30	66.30	68.40	49.80	83.70	65.20	1427	67.70
LaneATT	ResNet18	91.17	72.71	65.82	68.03	49.13	87.82	63.75	1020	68.58
	ResNet34	92.14	75.03	66.47	78.15	49.39	88.38	67.72	1330	70.72
	ResNet122	91.74	76.16	69.47	76.31	50.46	86.29	64.05	1264	70.81
LaneAF	ERFNet	91.10	73.32	69.71	75.81	50.62	86.86	65.02	1844	70.90
	DLA34	91.80	75.61	71.78	79.12	51.38	86.88	72.70	1360	73.03
SGNet	ResNet18	91.42	74.05	66.89	72.17	50.16	87.13	67.02	1164	70.67
	ResNet34	92.07	75.41	67.75	74.31	50.90	87.97	69.65	1373	72.69
FOLOLane	ERFNet	92.70	77.80	75.20	79.30	52.10	89.00	69.40	1569	74.50
CondLane	ResNet18	92.87	75.79	70.72	80.01	52.39	89.37	72.40	1364	73.23
	ResNet34	93.38	77.14	71.17	79.93	51.85	89.89	73.88	1387	73.92
	ResNet101	93.47	77.44	70.93	80.91	54.13	90.16	75.21	1201	74.80
CLRNet	DLA34	93.73	79.59	75.30	82.51	54.58	90.62	74.13	1155	75.37
CCCNet (ours)	DLA34	93.83	78.46	77.06	82.55	54.95	90.57	75.29	1240	76.01

**Fig 5:**
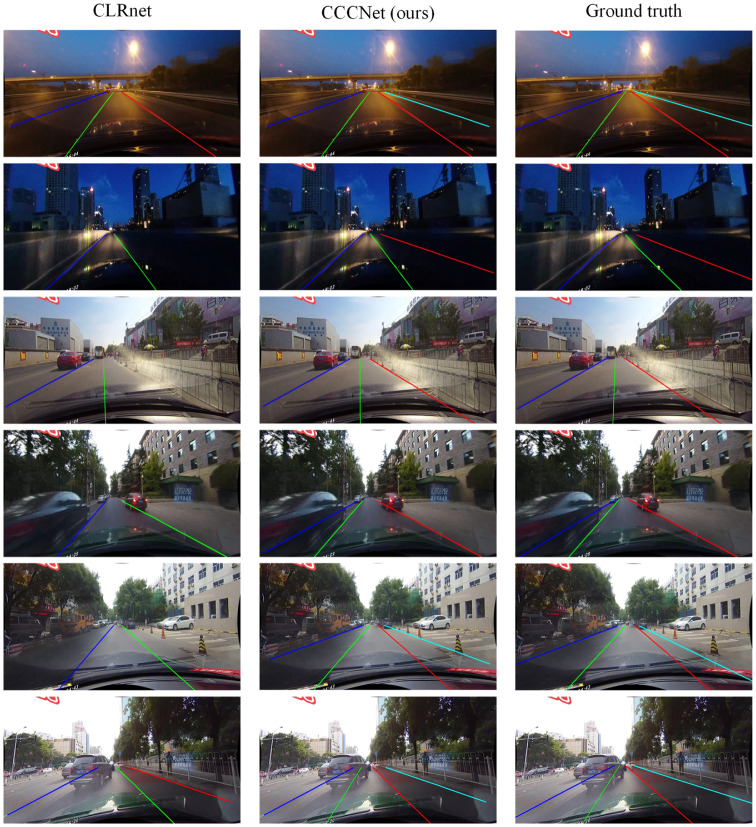
Comparison of experimental results on CULane dataset.

In order to provide a clear illustration of the enhanced lane detection performance of the CCCNet model, the test results on the CULane dataset have been visualized. Fig 5 provides a comparative visualization of the test outcomes from both CCCNet and CLRNet under identical conditions, with the ground truth provided for reference. This figure depicts lane detection in challenging situations like shadows, nighttime, and Dazzle scenarios. It is evident from the comparison with the ground truth that CLRNet could not detect all lane lines in these situations, while CCCNet successfully identified all lane lines. These experimental results robustly demonstrate the superiority of CCCNet in lane detection, particularly in complex scenarios. By effectively capturing global contextual information and mitigating the interference of irrelevant information, CCCNet achieves enhanced accuracy and robustness in lane detection tasks.

Table 3 displays the test results of our proposed CCCNet compared to other leading lane detection models, such as CLRNet, using the Tusimple dataset. The optimal performances are emphasized in bold, with the primary evaluation metric being F1. A quick examination of the table reveals marginal disparities in F1 scores among the later models, indicating a plateau in lane detection performance on the Tusimple dataset. However, our CCCNet consistently delivers improved results, achieving an SOTA result of 97.93.

**Table 3:** Comparison of experimental results on Tusimple dataset.

Method	Backbone	F1(%)	Acc(%)	FP(%)	FN(%)
SCNN	VGG16	95.97	96.53	6.17	1.80
RESA	ResNet34	96.93	96.82	3.63	2.48
PolyLaneNet	EfficientNetB0	90.62	93.36	9.42	9.33
E2E	ERFNet	96.25	96.02	3.21	4.28
UFLD	ResNet18	87.87	95.82	19.05	3.92
	ResNet34	88.02	95.86	18.91	3.75
LaneATT	ResNet18	96.71	95.57	3.56	3.01
	ResNet34	96.77	95.63	3.53	2.92
	ResNet122	96.06	96.10	5.64	2.17
FOLOLane	ERFNet	96.59	96.92	4.47	2.28
CondLaneNet	ResNet18	97.01	95.48	2.18	3.80
	ResNet34	96.98	95.37	2.20	3.82
	ResNet101	97.24	96.54	2.01	3.50
CLRNet	ResNet18	97.89	96.84	2.28	1.92
	ResNet34	97.82	96.87	2.27	2.08
	ResNet101	97.62	96.83	2.37	2.38
CCCNet(ours)	ResNet18	97.93	96.83	2.08	2.05
	ResNet34	97.84	96.83	2.27	2.04
	ResNet101	97.67	96.69	1.90	2.77

### Ablation experiment

Table 4 highlights the effectiveness of CCA in feature extraction for lane line detection. We compared our proposed model, CCCNet, with the state-of-the-art (SOTA) model, CLRNet, which does not fully leverage CCA information—a crucial factor for achieving high performance. By effectively utilizing CCA, our CCCNet model enhances the performance of lane line detection tasks. The experiments were validated on the CULane dataset, with various scenarios selected as evaluation metrics to analyze the detection results. As demonstrated in Table 4, the CCCNet model, which efficiently incorporates CCA information, outperforms CLRNet in most evaluation metrics.

**Table 4:** Ablation experiment.

Method	Backbone	CCA	Normal	Crowded	Dazzle	Shadow	No line	Arrow	Curve	Cross	Night
CLRNet	DLA34		93.73	**79.59**	75.30	82.51	54.58	**90.62**	74.13	**1155**	75.37
CCCNet(ours)	DLA34	*	**93.83**	78.46	**77.06**	**82.55**	**54.95**	90.57	**75.29**	1240	**76.01**

## Conclusion and discussion

In this study, we introduced CCCNet, an advanced lane detection model that significantly enhances the capabilities of autonomous driving systems by addressing the limitations of the CLRNet architecture. Our key contribution is the integration of the CCA as a global attention mechanism within the FPN of CLRNet, which optimizes the capture of contextual information and improves accuracy and robustness in challenging scenarios such as shadows, nighttime, obscured or highlighted lanes, and high lighting conditions.

Comprehensive evaluations on the CULane and TuSimple datasets demonstrate the superior performance of CCCNet over CLRNet and other mainstream models. On the CULane dataset, CCCNet achieved state-of-the-art results, with notable improvements in challenging scenarios: in the Dazzle scenario, performance improved from 75.30 to 77.06, and in the Night scenario, it increased from 75.37 to 76.01. Additionally, CCCNet showed a slight advantage in mF1 and significant gains in F1@50 and F1@75 metrics across the entire test set. On the TuSimple dataset, CCCNet achieved an SOTA F1 score of 97.93, consistently outperforming other models despite the marginal disparities observed in later models, indicating a performance plateau.

CCCNet shows notable improvements in lane detection accuracy and robustness but has limitations, particularly concerning computational complexity due to the cross-attention mechanism, which hinders real-time applicability in resource-constrained environments like autonomous vehicle embedded systems. This may necessitate optimization techniques such as model pruning or quantization. Despite strong performance on benchmark datasets like CULane and TuSimple, CCCNet may struggle with generalization to unseen scenarios, such as unusual lane markings or extreme weather, highlighting the need for diverse training data and domain adaptation techniques. It also faces challenges in extreme conditions like heavy rain or dense fog and depends heavily on high-quality data; inadequate or biased datasets can lead to suboptimal performance, potentially mitigated by active learning or synthetic data generation. Lastly, while accurate, CCCNet’s real-time performance on hardware with limited computational resources needs further optimization through techniques like model distillation or hardware acceleration. Addressing these limitations is crucial for deploying CCCNet in real-world autonomous driving systems and will guide future research.
